# Impact of the time to achieve viral control on the dynamics of circulating HIV-1 reservoir in vertically infected children with long-term sustained virological suppression: A longitudinal study

**DOI:** 10.1371/journal.pone.0205579

**Published:** 2018-10-23

**Authors:** Matías Moragas, Maximiliano Distefano, Debora Mecikovsky, Solange Arazi Caillaud, Carolina Cernadas, Rosa Bologna, Paula Aulicino, Andrea Mangano

**Affiliations:** 1 Laboratorio de BiologíaCelular y Retrovirus-CONICET, Hospital de Pediatría “Prof. Dr. Juan P. Garrahan”, Ciudad Autónoma de Buenos Aires, Argentina; 2 Servicio de Epidemiología e Infectología, Hospital de Pediatría “Prof. Dr. Juan P. Garrahan”, Ciudad Autónoma de Buenos Aires, Argentina; 3 DirecciónAsociada de Docencia e Investigación, Hospital de Pediatría “Prof. Dr. Juan P. Garrahan”, Ciudad Autónoma de Buenos Aires, Argentina; University of Pittsburgh, UNITED STATES

## Abstract

**Objective:**

Determine the decay rate of HIV-1 DNA reservoir in vertically infected children during sustained viral suppression (VS) and how it is affected by the age at VS.

**Methods:**

This study included 37 HIV-1 vertically infected children on suppressive antiretroviral therapy for at least 4 years. Children were grouped according to the age of antiretroviral therapy initiation (≤0.5 or >0.5 yrs) and to the age at VS (≤1.5, between >1.5 and 4, and >4 years). Decay of cell-associated HIV-1 DNA (CA-HIV-DNA) level and 2-long terminal repeats (2-LTR) circles frequency were analyzed over 4 years of viral suppression using piecewise linear mixed-effects model with two splines and logistic regression, respectively.

**Results:**

CA-HIV-DNA in peripheral blood mononuclear cells had a significant decay during the first two years of VS [-0.26 (95% CI: -0.43, -0.09) log_10_ copies per one million cells (cpm)/year], and subsequently reached a plateau [-0.06 (95% CI: -0.15, 0.55) log_10_ cpm/year]. The initial decay was higher in children who achieved VS by 1.5 years of age compared to those who achieved VS between >1.5 and 4 years and those after 4 years of age: -0.51 (95% CI:-0.94, -0.07), -0.35 (95% CI:-0.83, 0.14), and -0.21 (95% CI:-0.39, -0.02) log_10_cpm PBMC/year, respectively. The 2-LTR circles frequency decayed significantly, from 82.9% at pre-VS to 37.5% and 28.1% at 2 and 4 years of VS, respectively (P = .0009).

**Conclusions:**

These data highlight that achieving VS during the first 18 months of life limit the establishment of HIV-1 reservoirs, reinforcing the clinical benefit of very early effective therapy in children.

## Introduction

Although antiretroviral therapy (ART) allows sustained viral suppression (VS) in blood plasma, the ability to eradicate human immunodeficiency virus type 1 (HIV-1) has not been yet achieved. The principal obstacle to viral eradication is the quick establishment of HIV-1 in its target cells, mainly in resting memory CD4^+^ T cells, at the earliest phases of primary infection [1–3]. The half-life of these resting cells can reach up to several years and contribute to the persistence of the infection, being recognized as the principal HIV-1 reservoir [4–6].

More than 150,000 new pediatric HIV-1 infections occurred globally during 2015. Mother-to-child transmission is still the main route of infection in children, despite the extended prophylaxis regimen: during pregnancy, intrapartum, and the first six weeks after birth [7]. Initially, ART was recommended only for children with acquired immunodeficiency syndrome-related symptoms (clinical stage C) or severe immune suppression (stage 3) [8]. However, in recent years, many studies reported that early initiation of ART in all HIV-1-infected children has clinical benefits [9,10]. Current HIV-1 treatment guidelines strongly recommend ART initiation as soon as possible after positive HIV-1 diagnosis, regardless of clinical and immunological conditions [11,12]. This counterpart to this scenario is that most HIV-1 infected infants do not have the opportunity to start ART soon after birth, drugs are inaccessible for many patients, and there are side effects associated with prolonged ART use [13]. Simplified ART regimens, including break periods or interruption schedules, are being studied in order to improve treatment adherence and reduce the toxic effects associated with the drugs [14,15]. These new therapeutic approaches that include HIV-1 infected patients with low to undetectable plasma viral load (pVL) require ongoing monitoring of the HIV-1 reservoir size. Estimation of the reservoir size could be important to determine patient response and avoid the possibility of pVL rebound. An effective biomarker to measure reservoir size and residual ongoing viral replication has not been established; however, cell-associated HIV-1 DNA (CA-HIV-DNA) and 2-long terminal repeat (2-LTR) circles levels, respectively, are commonly used for these purposes [16]. CA-HIV-DNA may remain detectable despite sustained undetectable pVL; thus, its level of decay might be of interest when assessing the long-term efficacy of ART on reservoirs. However, it is not well defined how the time to achieve sustained VS, particularly in those infants who controlled virus replication at chronic stage of infection, impacts on the size of the HIV-1 reservoir.

The present study was conducted to further understand how the time to achieve effective ART response affects the persistence of HIV-1 in children throughout successfully suppressed viremia. The specific aims were to determine the decay of viral reservoirs, estimated by CA-HIV-DNA and 2-LTR circles, during long-term VS in children initiating ART during acute and chronic stages of HIV-1 infection, and to assess the impact of the timing to achieve viral suppression on subsequent CA-HIV-DNA and 2-LTR circles decay.

## Materials and methods

### Study cohort

**Fig 1:**
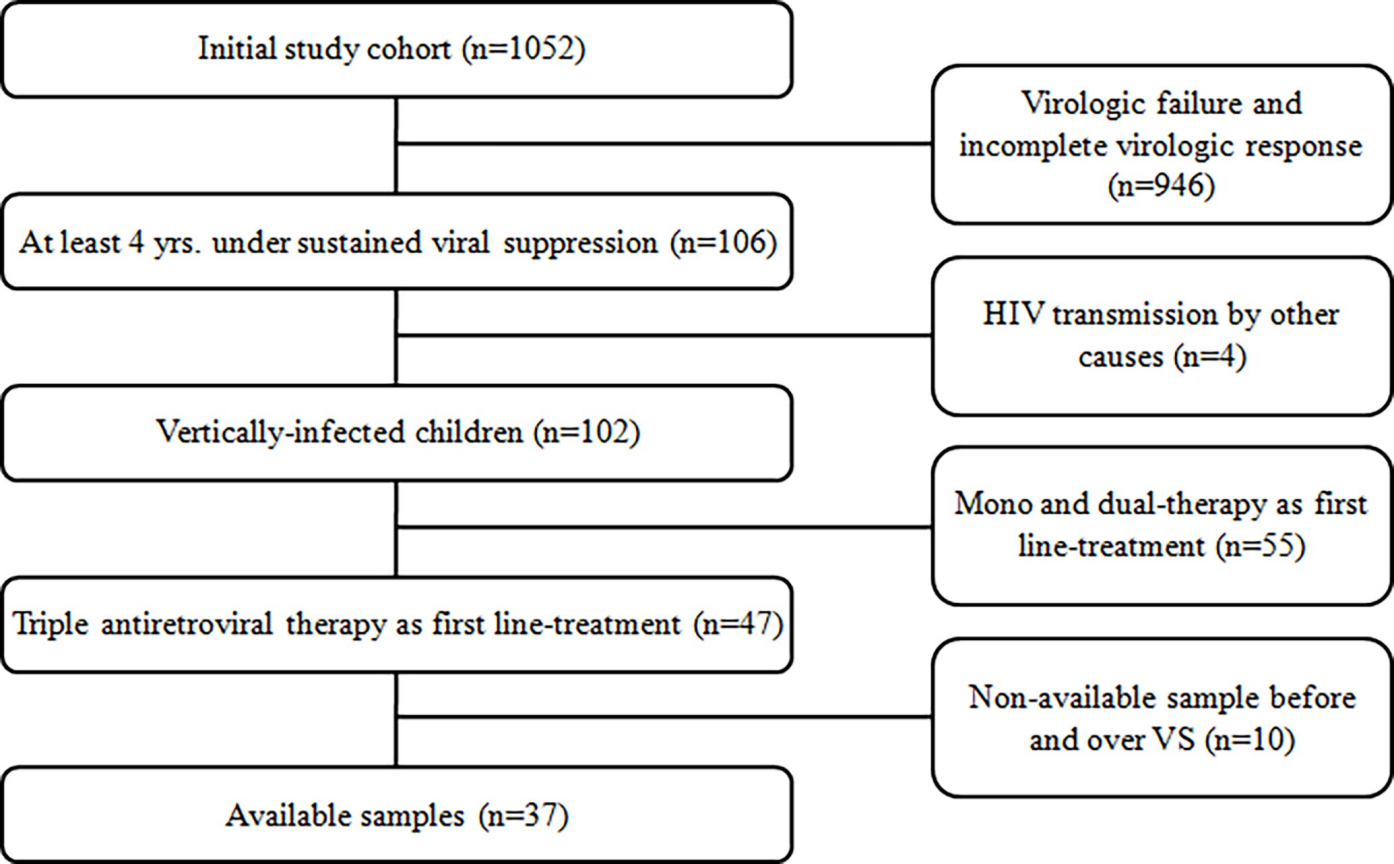
Study population. Newly HIV-1 infected children detected in Garrahan Pediatric Hospital from 1900 to 2008.

The initial cohort included 1,052 HIV-1 infected children who were treated at Hospital de Pediatría "Prof. Dr. Juan P. Garrahan", a referral pediatric hospital in Buenos Aires, Argentina. From this cohort, a subset of 37 patients born between 1990 and 2008 were selected for analysis based on the following inclusion criteria: I) HIV-1 acquired by vertical transmission; II) VS present for ≥4 years; III) first ART regimen based on effective triple therapy, including two nucleoside reverse transcriptase inhibitors (NRTIs) plus one non-nucleoside reverse transcriptase inhibitor (NNRTI) or protease inhibitor (PI); and IV) at least 2 available peripheral blood mononuclear cell (PBMC) or DNA samples for reservoir size quantification before ART initiation(pre-VS), and during 4 years of VS (Fig 1). Achieving two consecutive pVL below the limit of quantification (LoQ) of the available assay (range: 37–400 copies/mL) was defined as the beginning of sustained VS, and also maintaining this level along the study was defined as the maintenance of VS. Intermittent “blips” in the pVL after achieving VS were allowed, with blips defined as detectable pVL lower than 1000 copies of HIV-1 RNA/mL in one sample followed by undetectable levels. Patients were stratified according to the age atART initiation (≤0.5 or >0.5 yrs) and age to achieve VS (≤1.5, between >1.5 and 4, and >4 years). The study was approved by the Institutional Review Board (IRB) and Ethics Committee (EC) of Garrahan Pediatric Hospital (Protocol number: 856/2015). Written informed consent was obtained from parents or legal guardians. The consent process was approved by the IRB and EC.

For each patient included in the study, immunological data (CD4^+^cell, CD8^+^ T cell, PBMC and monocyte counts), treatment history, and HIV-1 pVL data were collected.

### CA-HIV-DNA quantification and 2-LTR circles detection

Genomic DNA was extracted using QIAamp DNA Blood Mini Kit (Qiagen, Hilden, Germany) from PBMC following the manufacturer’s protocols. DNA samples were stored at −20°C until use. The quantification of HIV-1 reservoirs, estimated by CA-HIV-DNA, was performed by semi-nested, real-time polymerase chain reaction (PCR) as previously described [17], with modification for amplification of ltr-gag region. A combination of previously published [18] primers-probe sets with newly designed ones based on subtype B, F, and B/F recombinant strains from the Los Alamos National Laboratory database were used. The LoQ of the assay was 0.78 log_10_ copies per one million (cpm) cells. B-globin quantification by real-time PCR was used to determine the number of PBMC present in each reaction.

Considering that variations of CD4^+^ T cell counts during HIV-1 infection may induce profound differences in CA-HIV-DNA levels, PBMC and monocyte counts through hemogram and CD4^+^ T cells counts by fluorescence-activated cell sorting were used to express CA-HIV-DNA levels in different units, as previously reported by Avettand-Fènoël et al [19]: log_10_ cpm PBMC, log_10_ cpm CD4^+^ T cells, and log_10_ copies/mL whole blood.

The 2-LTR circle was amplified and detected by hemi-nested real-time PCR using SYBR Green and a primer set targeting a conserved region of LTR-U5 and LTR-U3. A combination of previously published [18] primers with newly designed ones were used.

### Statistical analysis

The trajectories of CA-HIV-DNA in PBMC, in the proportion of CD4^+^ T cells, and in whole blood over 4 years of VS were estimated by a piecewise linear mixed-effects model with a random slope for each child. The model included one change of slope at 2 years of VS based on the minimization of the Akaike information criterion and the immune population changes over the child’s age. In addition, time at VS and the presence of blips, both expressed as categorical variables, were included within the model to analyze their impact on CA-HIV-DNA levels in PBMC over the study period. ART regimen (grouped into NNRTI or PI) at VS and the basal pVL were tested to estimate the CA-HIV-DNA trajectories; however, they did not improve the model fit and were therefore not included in the final models. The frequency of 2-LTR circles following VS was studied using a generalized linear model -a logistic regression for repeated-measures- with the inclusion of CA-HIV-DNA level as a continuous variable.

The Mann-Whitney test and Kruskal-Wallis test were used to compare CA-HIV-DNA levels between two and more than two groups, respectively. Spearman's correlation coefficient was used to evaluate the associations between CA-HIV-DNA levels reported in PBMC and those reported in CD4^+^ T cells and whole blood.

Statistical analyses were performed with R, version 3.3.1 (R Foundation for Statistical Computing, Vienna, Austria).

## Results

### Patient characteristics

Baseline characteristics are reported in Table 1. Among the 37 children studied, the median age at ART was 2.4 [interquartile range (IQR), 0.8–5.1] years. Seven children started ART before 6 months of infection (IQR, 0.3–0.5 yrs) and 30 children after that time (IQR, 1.8–6.9 yrs). The majority of the children experienced a late effective response to ART, with a median age to achieve VS of 5.3 (IQR, 3.8–10.8) years. Only 4 (10.8%) children reached VS before 1.5 years, 6 (16.2%) children between >1.5 and 4 years, and 27 (73%) children after 4 years of age. In addition, 18 (48.6%) children had one or two blips during VS.

**Table 1:** Patient characteristics.

Characteristic	Value
Subjects, No.	37
Sex, No. (%), Female	19 (51.4)
ACTG 076 prophylaxis regimen, No. (%)^a^	
- None	24 (64.9)
- Partial	5 (13.5)
- Complete	5 (13.5)
- Unknown	3 (8.1)
Age at ART initiation, median (IQR), yrs	2.4 (0.8–5.1)
First ART regimen, No. (%)	
- 2 NRTIs + NNRTI	16 (43.2)
- 2 NRTIs + PI	21 (56.8)
Age at VS, median (IQR), yrs	5.3 (3.8–10.8)
ART regimen at VS, No. (%)	
- 2 NRTIs + NNRTI	23 (62.2)
- 2 NRTIs + PI	14 (37.8)
Time from ART initiation to VS, median (IQR), yrs	2.1 (0.5–4.1)
pVL at pre-VS, median (IQR), log_10_ copies/mL	5.89 (5.03–6.25)
Immunologic population at pre-VS	
- CD4^+^ T cell counts, median (IQR), %	19 (13–27)
- CD4^+^ T cell counts, median (IQR), cells/mm^3^	788 (325–1494)
- CD4:CD8 ratio, median (IQR)	0.49 (0.39–0.67)
Immunologic population at end point	
- CD4^+^ T cell count, median (IQR), %	34 (30–39)
- CD4^+^ T cell count, median (IQR), cells/mm^3^	956 (691–1130)
- CD4:CD8 ratio, median (IQR)	1.00 (0.78–1.29)

^a^ Classification criteria based on ACTG 076: Complete, zidovudine administration to HIV-1 infected women during pregnancy and to the child during the first six weeks of life; Partial, zidovudine administration only to the HIV-1-infected pregnant woman or to the child; None, no zidovudine administration.

Abbreviations: ART, antiretroviral therapy; IQR, interquartile range; NNRTIs, non-nucleoside reverse transcriptase inhibitors; NRTI, nucleoside reverse transcriptase inhibitor; PI, protease inhibitor; pVL, plasma viral load; VS, viral suppression.

### Cell-associated HIV-1 DNA kinetics

**Fig 2:**
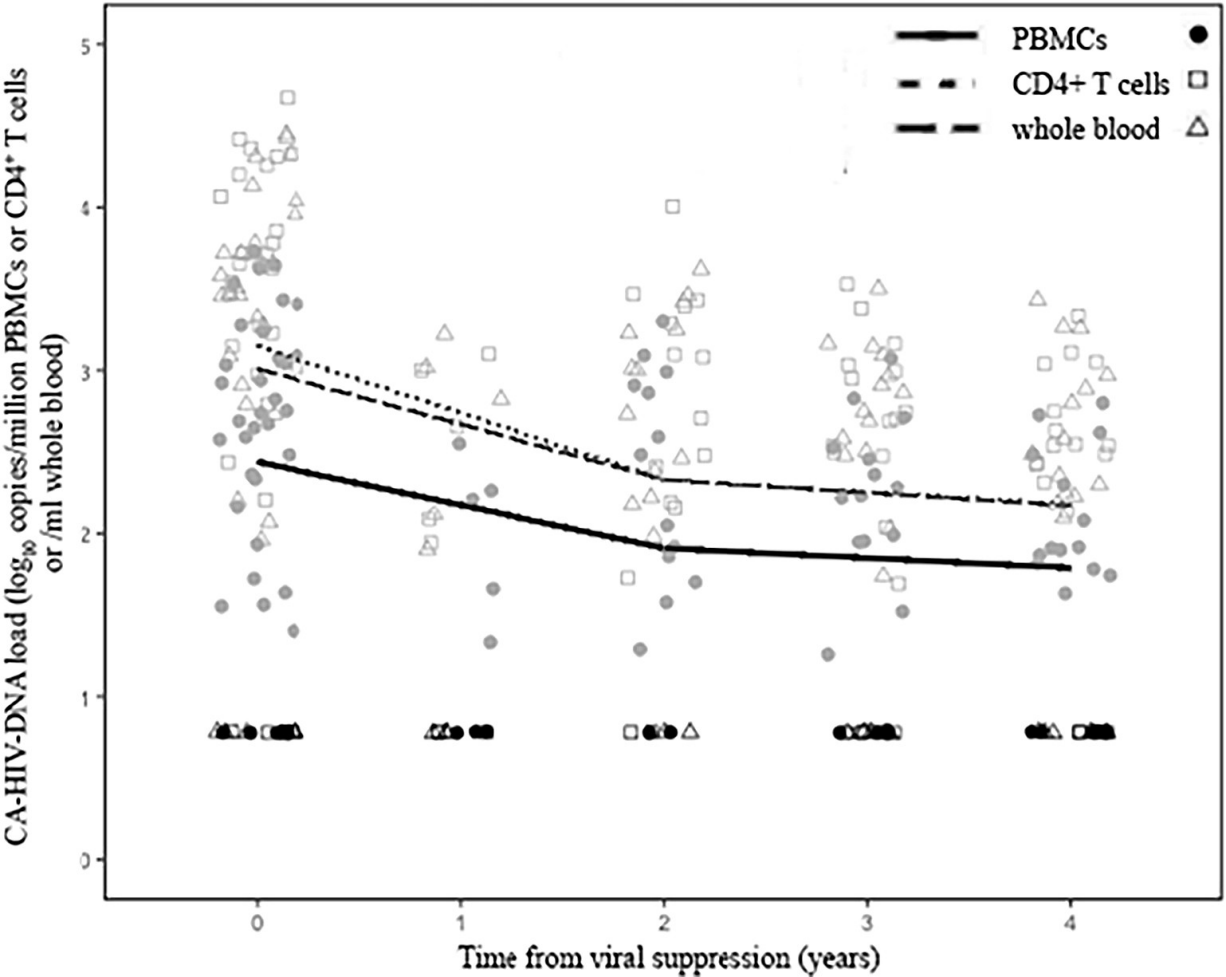
Decay rates of CA-HIV-DNA from children with sustained viral suppression according to the compartment analyzed. Individual lines indicate the trajectories of CA-HIV-DNA based on the model prediction. Individual points represent the quantification of HIV-1 DNA from each patient at specific time points. Levels of CA-HIV-DNA expressed as log_10_ cpm PBMC for PBMC, log_10_ cpm CD4^+^ T cells for CD4^+^ T-cells, and log_10_ copies/mL whole blood for whole blood.

The size of peripheral blood HIV-1 reservoirs was estimated by the level of CA-HIV-DNA. Among all the patients studied, the CA-HIV-DNA level was below the LoQ at pre-VS in only 5 children; this level persisted over the study period in 3 children, while remaining 2 children experienced a sustained increment in the CA-HIV-DNA level during VS. ThroughoutVS, 26 (70.3%) patients had quantifiable CA-HIV-DNA levels, and 11 (29.7%) patients had at least one CA-HIV-DNA measure below LoQ. The rate of decay and predicted mean level of CA-HIV-DNA estimated in PBMC, CD4^+^ T cells, and whole blood during VS are detailed in Table 2 and illustrated in Fig 2. The level of CA-HIV-DNA decayed significantly during the first two years of VS in all compartments, but it was more pronounced in CD4^+^ T cells than in whole blood and in PBMC: -0.41 [95% confidence interval (CI), -0.64 to -0.17] log_10_cpm CD4^+^/year, -0.34 (95% CI, -0.57 to -0.10) log_10_ copies/mL whole blood/year, and -0.26 (95% CI, -0.43 to -0.09) log_10_cpm PBMC/year, respectively. However, the decay rate of CA-HIV-DNA after 2 years of VS was reduced and was not statistically significant in all compartments: -0.08 (95% CI, -0.14 to 0.80) log_10_cpm CD4^+^/year, -0.08 (95% CI, -0.79 to 0.63) log_10_ copies/mL whole blood/year, and -0.06 (95% CI, -0.15 to 0.55) log_10_cpm PBMC/year.

**Table 2:** Estimated decay rates and predicted means of cell-associated HIV-1 DNA level over 4 years of sustained viral suppression and expressed in different units.

Variable	PBMCs	CD4^+^cells	Whole Blood
No. of measurements	98	92	93
Intercept^a^			
- Estimated (95% CI)	2.44 (2.17 to 2.70)	3.15 (2.79 to 3.50)	3.01 (2.66 to 3.35)
First slope (0–2 yrs)^b^			
- Estimated (95% CI)	-0.26 (-0.43 to -0.09)	-0.41 (-0.64 to -0.17)	-0.34 (-0.57 to -0.10)
- P-value^c^	.0028	.0009	.0056
Second slope (2–4 yrs)^b^			
- Estimated (95% CI)	-0.06 (-0.15 to 0.55)	-0.08 (-0.14 to 0.80)	-0.08 (-0.79 to 0.63)
- P-value^c^	.2578	.1619	.2842
Level of CA-HIV-DNA^d^			
- At 0 yrs of VS, mean (95% PI)	2.55 (2.18 to 2.93)	3.35 (2.82 to 3.88)	3.16 (2.64 to 3.68)
- At 2 yrs of VS, mean (95% PI)	2.03 (1.79 to 2.27)	2.53 (2.23 to 2.84)	2.49 (2.20 to 2.78)
- At 4 yrs of VS, mean (95% PI)	1.98 (1.66 to 2.30)	2.38 (1.96 to 2.81)	2.38 (1.97 to 2.80)

^a^The intercept represents the estimated basal level of CA-HIV-DNA and is expressed in different units by column: log_10_ cpm PBMC, log_10_ cpm CD4^+^ T-cells and log_10_ copies/mL whole blood.

^b^Slopes represent the decay rate of CA-HIV-DNA at different ages of VS and are expressed in different units by column: log_10_ cpm PBMC/year, log_10_ cpm CD4^+^ T-cells/year, and log_10_ copies/mL whole blood/year.

^c^P-value represents the statistical significance of each decay rate in each compartment estimated by piecewise linear mixed-effects model.

^d^Levels are expressed in different units by column: log_10_ cpm PBMC, log_10_ cpm CD4^+^ T-cells and log_10_ copies/mL whole blood. The 95% PIs were predicted from the final model.

Abbreviations: CA-HIV-DNA, cell-associated HIV-1 DNA; CI, confidence interval; PBMC, peripheral blood mononuclear cell; PI, prediction interval; VS, viral suppression.

The predicted mean level of CA-HIV-DNA in PBMC was 2.55 [95% prediction interval (PI), 2.18 to 2.93] log_10_cpm PBMC at VS and decayed to 2.03 (95% PI, 1.79 to 2.27) and 1.98 (95% PI, 1.66 to 2.30) log_10_cpm PBMC at 2 and 4 years of VS, respectively. These levels were lower than those expressed in the proportion of CD4^+^ T cells and whole blood. However, CA-HIV-DNA levels in PBMC were highly correlated with those levels in CD4^+^ T cells (r = 0.97, P < .0001) and in whole blood (r = 0.98, P < .0001).

Moreover, we observed a similar rate of decay in CA-HIV-DNA in PBMC between 2 and 4 years of VS in children who experienced the emergence of blips in comparison with those without blips: -0.02 (95% CI, -0.57 to 0.53) and -0.10 (95% CI, -0.63 to –0.44) log_10_cpm PBMC/year, respectively (P = .5702).

We further examined the effect of the age of infection at ART initiation on the level of CA-HIV-DNA in PBMC at 2 and 4 years of VS. The median level of CA-HIV-DNA in infants who started ART before 6 months of infection was 1.96 (IQR, 1.66–2.17) and 1.90 (IQR, 1.15–2.23) log_10_cpm PBMC at 2 and 4 years of VS, respectively, while infants who initiated ART afterward had a median level of 1.96 (IQR, 1.30–2.78) and 1.91 (IQR, 0.90–2.48) log_10_cpm PBMC at 2 and 4 years of VS, respectively. No significant difference was observed in the level of CA-HIV-DNA according to the time at ART initiation at 2 and 4 years of VS (P = .7632 and P = .5701, respectively).

**Fig 3:**
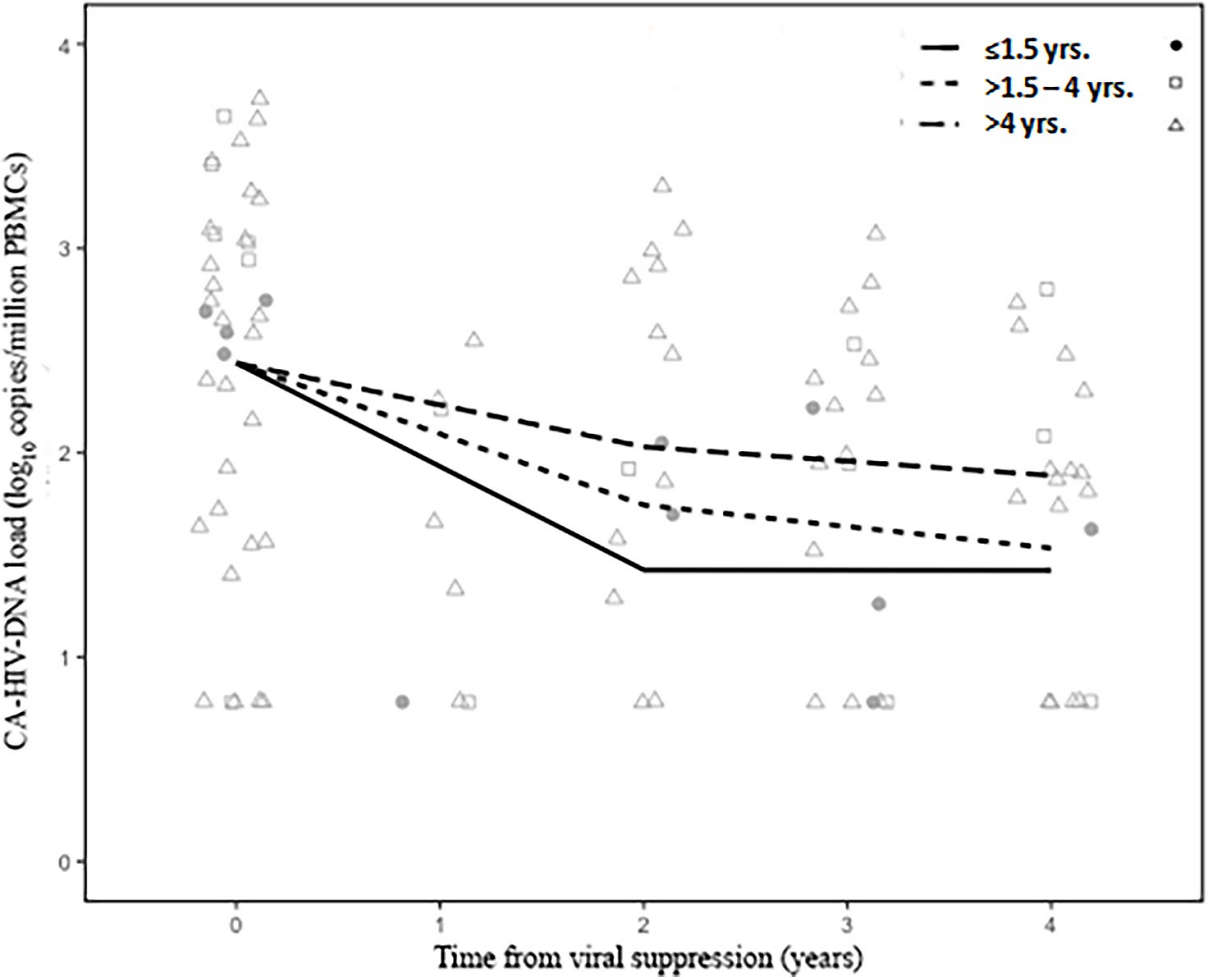
Decay rates of CA-HIV-DNA from children with sustained viral suppression according to the age of infection at VS. Individual lines indicate the trajectories of CA-HIV-DNA based on the model prediction. Individual points represent the quantification of HIV-1 DNA from each patient at specific time points.

Finally, we analyzed the decay rates of CA-HIV-DNA according to the age of infection at VS (≤1.5, >1.5–4, and >4 years). These results are shown in Table 3 and Fig 3. Slopes from children who achieved VS by 1.5 years of ageand from those who achieved VS after 4 years of age were significantly negative (P = .0235, P = .0287, respectively) during the first 2 years of VS. At that time, a higher decay rate of CA-HIV-DNA was observed in children who achieved VS by 1.5 years of age in comparison to those who achieved VS between >1.5 and 4 years and after 4 years of age: -0.51 (95% CI, -0.94 to -0.07), -0.35 (95% CI, -0.83 to 0.14), and -0.21 (95% CI, -0.39 to -0.02) log_10_cpm PBMC/year, respectively. However, the overall difference among these slopes was not statistically significant (P = .1645). After 2 years of VS, CA-HIV-DNA decay rate (second slope) was very slow and similar among all groups, with no statistically significant value in any group (P = .7346). Furthermore, the predicted mean level of CA-HIV-DNA after 4 years of VS tended to be lower in children who achieved VS before 1.5 years of age compared to those with VS between >1.5 and 4 years and those after 4 years of age: 1.56 (95% PI, 0.75 to 2.37), 1.83 (95% PI, 0.94 to 2.73), and 2.08 (95% PI, 1.73 to 2.43) log_10_cpm PBMC, respectively (P = .1942).

**Table 3:** Estimated decay rates and predicted means of cell-associated HIV-1 DNA level in PBMC over 4 years of sustained viral suppression according to theage at viral control.

	Age at VS			
Variable	≤1.5 yrs	>1.5–4 yrs	>4 yrs	P-value^d^
No. of children	4	6	27	NA
Intercept^a^				
- Estimated (95% CI)	2.44 (2.18 to 2.70)	2.44 (2.18 to 2.70)	2.44 (2.18 to2.70)	NA
First slope (0–2 yrs)^b^				
- Estimated (95% CI)	-0.51 (-0.94 to -0.07)	-0.35 (-0.83 to 0.14)	-0.21 (-0.39 to -0.02)	.1645
- P-value^c^	.0235	.1583	.0287	
Second slope (2–4 yrs)^b^				
- Estimated (95% CI)	-0.001 (-1.50 to 1.50)	-0.11 (-1.64 to 1.44)	-0.07 (-0.63 to 0.49)	.7346
- P-value^c^	.3477	.6495	.4798	
Level of CA-HIV-DNA (log_10_ cpm PBMC)				
- At 0 yrs of VS, mean (95% PI)	2.73 (2.01 to 3.45)	2.58 (1.86 to 3.30)	2.52 (2.13 to 2.90)	.5941
- At 2 yrs of VS, mean (95% PI)	1.72 (1.24 to 2.20)	1.89 (1.40 to 2.37)	2.11 (1.85 to 2.37)	.1614
- At 4 yrs of VS, mean (95% PI)	1.56 (0.75 to 2.37)	1.83 (0.94 to 2.73)	2.08(1.73 to 2.43)	.1942

^a^The intercept represents the estimated basal level of CA-HIV-DNA and is expressed in log_10_ cpm PBMC.

^b^Slopes represent the decay rate of CA-HIV-DNA at different ages of VS for each group and they are expressed in log_10_ cpm PBMC/year.

^c^P-value represents the statistical significance of each decay rate in each group estimated by piecewise linear mixed-effects model.

^d^P-value represents the statistical significance of the comparison among children with age at VS of ≤1.5, >1.5–4, and >4 years.

Abbreviations: CA-HIV-DNA, cell-associated HIV-1 DNA; CI, confidence interval; PBMC, peripheral blood mononuclear cell; PI, prediction interval; NA, not applicable; VS, viral suppression.

### Detection of 2-LTR circles and its association with CA-HIV-DNA levels

Among the 37 patients included in the study, the presence of 2-LTR circles was determined in 35 patients at pre-VS, in 24 patients at 2 years of VS, and in 32 patients at 4 years of VS. The frequency of children with detectable 2-LTR circles decayed over VS, from 82.9% (29/35) at pre-VS to 37.5% (9/24) and 28.1% (9/32) at 2 and 4 years of VS, respectively. The logistic regression analysis for repeated measures showed a significant decay in the frequency of 2-LTR circles detection during VS (P = .0009). No significant difference in 2-LTR circles detection at 4 years of VS was observed among the children grouped according to the age at VS (1/3 patients 1.5 years or earlier, 1/4 between >1.5 and 4 years and 7/25 after 4 years of age). Furthermore, children with detectable 2-LTR circles showed a higher median level of CA-HIV-DNA compared to those with undetectable 2-LTR circles [2.48 (IQR, 1.68–3.01) vs. 1.89 (IQR, 0.78–2.39) log_10_cpm, P < .005].

## Discussion

In this study, we evaluated the evolution of HIV-1 reservoir size on PBMC in vertically HIV-1-infected children. Our results showed a marked decay of HIV-1 DNA reservoir size during the first two years of VS, being higher in those children with earlier age at VS. Then, this decay reaches a plateau independently of the age at viral suppression.

Reduced size of HIV-1 reservoirs has been reported in adults and children who started ART at early stage of infection. However, there are controversial results regarding what time is early enough to limit reservoir size and also it depends on which cell subset is studied [20–23]. The kinetics of HIV-1 reservoirs has been well reported in adults, but there are few studies in children, mainly analyzing the immediate effect after ART initiation and with short follow-up [24]. Notwithstanding, the kinetics of HIV-1 reservoirs in pediatric population over a long term of VS and how they are affected by the age at VS are poorly defined. In this study, we address these issues by studying longitudinally children who achieved effective response to ART at different times. It is important to mention, that due to the retrospectively design of the study and the availability of store PBMCs samples, most of the children started ART later and some who started early had a delay to achieve viral suppression. Nowadays, following international guidelines HIV infected children start ART soon after diagnosis (mainly during the first year of life). However, in resource-limiting countries a delay in pediatric HIV diagnosis is still frequent with the consequently late initiation of therapy. Hence, exploring the dynamics of the viral reservoirs in these populations might bring insights of their behavior in well-established infection in children. Moreover, these patients might serve as good control groups for studies comparing the kinetics of viral reservoirs in children who start ART at early or very early steps of infection.

Our results showed a significant decay rate of CA-HIV-DNA level over the first two years of sustained VS. This was in concordance with previous studies performed in adults by Laanani et al [20] and Besson et al [25]. However, after 2 years of VS, the level of CA-HIV-DNA remained almost in a steady state. This plateau phase of CA-HIV-DNA might reflect ongoing viral replication or viral release from stable reservoirs [26–28]. Additionally, survival of long-lived HIV-1 infected cells could be another key factor contributing to the stability of the reservoir size, but their persistence during 4 years of VS is unlikely.

Activation of target cells and latently infected cells by concurrent infection or immune suppression by opportunistic infections might contribute to ongoing viral replication [29]. In our cohort, children with and without blips (48.6% vs 51.4%) had no difference in the decay rate of CA-HIV-DNA over VS, suggesting that a transitory viral rebound seems not to be enough to modify the whole size of HIV-1 reservoirs. In contrast, Ramratnam et al [30] observed that the rate of decay of HIV-1 reservoir is inversely correlated with the extent of residual viral replication during ART. Whether the time elapsed to recover VS since viral rebound occurred or if the highest level of viremia at rebound could modify reservoir size need to be addressed.

It is accepted that the earlier ART is initiated, the smaller the size of the HIV-1 reservoir [24].However, we did not observedifferences in the CA-HIV-DNA level after 4 years of VS between children starting ART before (n = 7) and after (n = 30) six months of age. This could be explained in part by the unbalanced number of children in both groups and also by a late effective viral control observed in our cohort despite early ART initiation. Furthermore, a recent study showed an absence of significant difference in CA-HIV-DNA levels during 96 weeks of ART among children who started ART before and after 6 weeks of life [31]. These findings suggest that the establishment of HIV-1 reservoirs occur very early—probably during the first hours after infection, that could be *in utero* as the Mississippi child [32].

We further analyzed whether there was an association between the age at VS and the decay rate of CA-HIV-DNA during VS. We found that the decay rate of CA-HIV-DNA during the first two years of VS tended to be higher in children who reached effective viral control by 1.5 years of age than in those beyond 1.5 years. Additionally, the predicted level of CA-HIV-DNA at 4 years of VS was lower when the time at VS was ≤1.5 years of age. In line with our findings, Persaud and colleagues reported, in a cross-sectional study, a significant difference in CA-HIV-DNA levels after 7.2 years of VS among children who suppressed plasma viremia before 1 year and after 5 years of age [33]. However, the authors did not find a significant difference between the groups who achieved VS before 1 year compared to 1–5 years or between the latter group and those after 5 years of age.

There are many controversies regarding data concerning the stability of 2-LTR circles and, in consequence, its value as a surrogate marker for ongoing replication. Some studies reported that 2-LTR circles are labile forms and thus their presence is indicative of recent infection events [34,35]. However, other studies indicated that these episomal HIV-1 forms are intrinsically stable in vitro [36,37]. In the present study, we found that, at ART initiation, even with high pVL (approximately 5 log_10_ copies/mL), 80% of the children had detectable levels of 2-LTR circles. This could reflect a methodological limitation since we isolated total DNA from PBMC instead of episomal DNA, which seems to improve 2-LTR circles detection [38]. Similar to the frequency observed with CA-HIV-DNA, the frequency of children with detectable 2-LTR circles significantly decreased over VS, and it was higher (28%) than it was reported by Koelsch et al [39] even at 4 years of VS. This level could be even higher taking into account the methodological limitation discussed above. Two main hypotheses might explain this: (1) ongoing viral replication during VS manifested by residual viremia allows de novo generation of HIV-1 episomal forms or (2) 2-LTR circles generated before suppressive ART persists for longer time than expected, remaining detectable even during many years of sustained VS in children.

Some limitations of the present study include the predominance of children with late effective response to ART, resulting in some groups analyzed being imbalanced; therefore, the decay of HIV-1 reservoirs in those groups might be underpowered. Also, the subjective data on ART adherence in our pediatric cohort could underestimate the impact of time at ART initiation on HIV-1 reservoirs.On the other hand, it is important to highlight that molecular DNA assays could overestimates the size of the reservoirs since they detect intact and defective viruses [16,40]

The high level and the low decay rate of CA-HIV-DNA during VS found in our study seem to be an obstacle for ART-free remission. However, it has been previously reported that more than 95% of CA-HIV-DNA is defective to produce replication-competent viruses [41,42]. In this sense, the adults ANRS VISCONTI cohort study [43] and, more recently, the French ANRS EPF-CO10 pediatric cohort [44] demonstrated that functional cure could be achieved without complete elimination of CA-HIV-DNA. In a recent, very novel study, Imamichi and colleagues [41] proposed that defective proviruses are capable of transcribing unspliced HIV-1 RNA producing intact HIV-related proteins in patients receiving ART who suppressed pVL for many years. Collectively, these findings lead to a new understanding of the role of CA-HIV-DNA in HIV-1 cure studies.

In conclusion, our data highlight the difficulty to eliminate the circulating reservoirs from peripheral blood. Also, our results support that the age to reach VS may be a better predictor of the size of HIV-1 reservoirs on PBMC in children after a prolonged time of VS than the age at ART initiation, reinforcing the clinical benefit of very early effective therapy.

## Supporting information

S1 Table Cohort data set. (XLSX)
